# Optimizing gastric cancer treatment: the role of LODDs in lymph node staging

**DOI:** 10.3389/fonc.2026.1828429

**Published:** 2026-06-09

**Authors:** Zhao Hao, Haiyan Niu, Yuebo Bi, Qimin Sun, Wenjun Yang

**Affiliations:** 1 Key Laboratory of Tropical Translational Medicine of Ministry of Education, School of Basic Medicine Sciences, Hainan Medical University, Haikou, China; 2 Department of Pathology, the First Affiliated Hospital, Hainan Medical University, Haikou, China

**Keywords:** gastric adenocarcinoma, lymph node staging, log odds of positive lymph nodes, lymph node ratio, prognosis, nomogram

## Abstract

**Background:**

Gastric cancer is one of the most common malignancies worldwide and is associated with poor prognosis, placing a considerable burden on public health. Overall treatment outcomes remain unsatisfactory, and accurate lymph node staging is essential for optimizing therapeutic strategies and improving survival. Although the prognostic value of log odds of positive lymph nodes (LODDs) has been investigated in gastric cancer, its comparative performance against AJCC N stage and lymph node ratio (LNR) in gastric adenocarcinoma, particularly with external institutional validation and sensitivity analyzes based on adequate lymph node evaluation, remains insufficiently characterized. This study aimed to compare the prognostic value of AJCC-N, LNR and LODDs in gastric adenocarcinoma and to develop an externally validated LODDs-based prognostic nomogram.

**Methods:**

We included 4,054 patients with gastric adenocarcinoma from the SEER database (2015–2019) who underwent gastrectomy and had available regional lymph node examination data, as well as 383 patients from the First Affiliated Hospital of Hainan Medical University who underwent gastrectomy with operatively and pathologically confirmed D2 lymphadenectomy. Because anatomical D2 lymphadenectomy status cannot be directly identified in SEER, examined lymph node count was used as an indicator of the adequacy of lymph node evaluation, and sensitivity analyzes were performed in SEER patients with ≥16 examined lymph nodes. Clinicopathological variables included sex, age, race, tumor size, T stage, AJCC N stage (AJCC-N), lymph node ratio (LNR), and log odds of positive lymph nodes (LODDs). The primary endpoint was overall survival (OS), defined as the time from the date of surgery to death from any cause or last follow-up. Between-group comparisons were performed using the chi-square test. Optimal cut-off values were determined with X-tile software. Survival differences were evaluated by Kaplan–Meier curves. Time-dependent receiver operating characteristic (ROC) curves and corresponding time-dependent areas under the curve (AUCs) were used to compare the predictive performance of AJCC-N, LNR and LODDs for 1-, 3- and 5-year OS. Cox regression models were applied to identify independent prognostic factors, which were then incorporated into a nomogram. Nomogram performance was assessed using calibration curves, time-dependent C-index analysis and decision curve analysis (DCA).

**Results:**

AJCC-N, LNR and LODDs were strongly and positively correlated in all three datasets (P < 0.001). Time-dependent ROC analysis showed that LODDs had slightly larger areas under the curve than LNR and AJCC-N for predicting 1-, 3- and 5-year survival. Multivariable Cox regression confirmed that LODDs, together with sex, age, race, T stage and tumor size, were independent risk factors for overall survival (P < 0.05). The nomogram constructed from these factors showed good agreement between predicted and observed outcomes on calibration curves, and DCA indicated meaningful clinical net benefit across a broad range of threshold probabilities.

**Conclusion:**

LODDs sensitively reflects metastatic tumor burden and showed favorable prognostic performance compared with AJCC-N and LNR in gastric adenocarcinoma. The LODDs-based nomogram may serve as a useful tool for individualized prognostic assessment, although further validation in well-defined lymphadenectomy cohorts is warranted.

## Introduction

Gastric cancer is a major malignant tumor that threatens human health, with high incidence and mortality worldwide and a substantial impact on global public health. Epidemiological data highlight the ongoing burden of this disease. Although the incidence of gastric cancer has shown a decreasing trend in recent years, it remains the fifth most common cancer and one of the leading causes of cancer-related death worldwide. In 2022, there were approximately 968,300 newly diagnosed cases of gastric cancer and around 659,800 deaths attributable to this disease (1, 2). These figures underscore the wide prevalence and serious impact of gastric cancer on a global scale. The severity of gastric cancer is also reflected in its complex biological behavior and generally poor prognosis. Gastric cancer is highly heterogeneous, with considerable variation in clinical presentation, treatment response and survival outcomes (3). Cancer stem cells (CSCs) are intrinsically resistant to conventional chemotherapy and radiotherapy, and they play an important role in tumor recurrence and metastasis (4). Despite advances in diagnostic methods and treatment strategies, overall outcomes remain unsatisfactory, and the 5-year survival rate of patients with gastric cancer is still relatively low (5). Challenges are particularly evident in patients with locally advanced or advanced disease, who often present with aggressive tumors and extensive metastasis, making treatment selection and prognostic evaluation more complex. A robust and accurate staging system is therefore crucial, as it can guide treatment decisions and support more individualized and effective therapeutic strategies.

Multiple studies have shown that different lymph node staging systems are valuable for prognostic evaluation and treatment guidance in patients with gastric cancer. For example, a hybrid lymph node staging system based on both anatomic location and the number of metastatic lymph nodes was proposed in a study of 6,025 patients who underwent gastrectomy for primary gastric cancer. That study found that, even within the same pN category, patients with higher stages in the new N classification had worse prognosis, suggesting that the anatomic extent of nodal metastasis is important and that the new system performs comparably to the current TNM classification for prognostic assessment, thus offering a possible alternative (6). Other work comparing three lymph node staging schemes—based on the number of metastatic lymph nodes, LNR, and LODDs—in patients who received D2 resection plus adjuvant chemotherapy showed that LNR provided the best discrimination of survival among patients with locally advanced gastric cancer (7). The number of examined lymph nodes is also critical for accurate staging and for improving survival. A cohort study of 8,696 patients with gastric cancer from the United States and China indicated that a higher number of examined lymph nodes was associated with more accurate staging and improved postoperative survival. That study suggested a minimum of 17 examined lymph nodes and an optimal number of 33 to assess the quality of nodal examination and stratify postoperative survival (8). Another analysis of 7,620 patients who underwent curative resection for gastric cancer confirmed the importance of increasing the number of examined nodes for accurate prognostic evaluation, recommending that more than 30 lymph nodes be examined in patients with positive nodes (9). Several newer staging systems have also shown promise. A modified pathological staging system based on LNR and the number of examined nodes has been reported to outperform the conventional AJCC staging system in prognostic assessment (10). In addition, a hybrid system that incorporates both lymph node location and number demonstrated better prognostic performance than the 8th edition AJCC pathological N staging in a training cohort of 2,598 patients and an external validation cohort of 756 patients (11).

The log odds of positive lymph nodes (LODDs) is a relatively new method of nodal staging that has shown prognostic value in several cancers. Multiple studies have demonstrated a close association between LODDs and survival across different tumor types. For example, in a study of 440 patients with intrahepatic cholangiocarcinoma (ICC) who underwent curative resection, LODDs was an independent risk factor for survival and was proposed as the optimal lymph node-based prognostic index for ICC (12). In patients undergoing R0 resection for pancreatic cancer, LODDs has also been identified as an independent prognostic factor, with better predictive performance than N stage and LNR among node-negative patients (13). Compared with conventional nodal staging methods, LODDs can reduce the influence of factors related to surgery, pathology, tumor characteristics and host status on nodal assessment. For instance, when evaluating patients with adenocarcinoma of the esophagogastric junction, LODDs outperformed N stage, LNR and the number of negative lymph nodes in predicting prognosis among patients with nodal metastasis (14). In endometrial carcinosarcoma, a prognostic nomogram based on LODDs provided more accurate and convenient prediction of overall survival than the FIGO staging system (15). However, LODDs is not the optimal method in every cancer type. In gallbladder cancer, a prognostic model based on the number of metastatic lymph nodes showed higher accuracy than LODDs (16). In pancreatic cancer, a staging system based on the number of metastatic lymph nodes was reported as the most suitable prognostic indicator, with performance comparable to that of LODDs and LNR (17). Thus, the most appropriate lymph node staging method may differ across cancer types, and the combined use of multiple indices may help optimize individualized treatment strategies and improve survival.

Although LODDs has been previously evaluated in gastric cancer, including several SEER-based nomogram studies, existing studies have mainly focused on selected populations and have differed in patient selection, validation strategy and comparison with AJCC-N and LNR. Therefore, the incremental value of LODDs in surgically treated gastric adenocarcinoma remains worthy of further validation.

In this study, we used a large SEER cohort and an external institutional cohort to systematically compare AJCC-N, LNR and LODDs, construct a LODDs-based nomogram, and perform ELNs ≥16 sensitivity analyzes to evaluate the robustness of LODDs in patients with adequate lymph node evaluation.

## Materials and methods

### Study population

Clinical information on 24,610 patients diagnosed with gastric cancer and treated surgically between 2015 and 2019 was extracted from the SEER database. In parallel, data from 503 patients who underwent surgery for gastric cancer between 2015 and 2019 at the First Affiliated Hospital of Hainan Medical University were retrospectively collected.

For the SEER cohort, the inclusion criteria were as follows: age 18–75 years; histologically confirmed gastric adenocarcinoma; non-metastatic disease; gastrectomy as cancer-directed surgery; available information on the number of examined and positive regional lymph nodes; and complete survival information. Because the SEER database does not provide station-based operative information, anatomical D2 lymphadenectomy could not be directly ascertained in this cohort. Therefore, SEER patients were not defined as having undergone D2 lymphadenectomy.

For the institutional validation cohort, the inclusion criteria were as follows: age 18–75 years; histologically confirmed gastric adenocarcinoma; non-metastatic disease; gastrectomy with D2 lymphadenectomy; negative resection margins; and complete clinicopathological and follow-up information. D2 lymphadenectomy in the institutional cohort was confirmed by reviewing operative records and pathological reports.

The exclusion criteria for both cohorts were: palliative surgery or positive resection margins; non-adenocarcinoma histology; distant metastasis; and incomplete clinical or follow-up data. Patients with incomplete clinical variables or follow-up information were excluded using a complete-case approach, and no imputation was performed. Because of the retrospective nature of the datasets, the missing-data mechanism could not be fully determined.

After applying these criteria, 4,054 patients with gastric adenocarcinoma from the SEER database were included and randomly assigned to a training set and an internal validation set. A total of 383 patients from the First Affiliated Hospital of Hainan Medical University were included as an external validation cohort.

### Data collection and definitions

The following variables were collected: sex, age, race, tumor size, T stage, N stage, number of examined lymph nodes, number of positive lymph nodes, number of negative lymph nodes, LNR category and LODDs category. Pathological staging followed the 8th edition AJCC TNM classification.

The number of examined lymph nodes was defined as the total number of regional lymph nodes pathologically evaluated after surgery. The number of positive lymph nodes was defined as the number of regional lymph nodes with pathological metastasis. The number of negative lymph nodes was calculated as the number of examined lymph nodes minus the number of positive lymph nodes.

LNR and LODDs were defined as:

LNR = (number of positive lymph nodes)/(total number of examined lymph nodes)
LODDs = log [(number of positive lymph nodes + 0.5)/(number of negative lymph nodes + 0.5)]

Optimal cut-off values for LNR and LODDs were determined using X-tile software version 3.61 in the training cohort, and the same cut-off values were applied unchanged to the internal validation cohort, external validation cohort and ELNs ≥16 sensitivity subgroup. In the SEER cohort, examined lymph node count was used only as an indicator of the adequacy of lymph node evaluation, not as direct evidence of D2 lymphadenectomy.

### Follow-up

Follow-up was performed mainly through outpatient visits, telephone interviews and review of medical records or database information. Follow-up started on the date of surgery and continued until 31 December 2024. Patients were generally reviewed every 3–6 months during the first year after surgery, every 6–12 months thereafter, and once per year after 3 years of survival. The primary endpoint of this study was overall survival (OS), which was defined as the time from the date of surgery to the date of death from any cause or the date of last follow-up. Patients who were alive at the last follow-up were censored.

In the external validation cohort from the First Affiliated Hospital of Hainan Medical University, 35 patients were lost to follow-up, yielding a loss-to-follow-up rate of 9.1%. The last follow-up date was December 2024.

### Statistical analysis

All statistical analyzes and visualizations were performed using R software (version 4.3.2). Categorical variables were compared using the chi-square test. X-tile software was used to calculate optimal cut-off points for LNR and LODDs only in the training cohort. The same cut-off values were then applied to the internal validation cohort, external validation cohort and ELNs ≥16 sensitivity subgroup without further optimization. Kaplan–Meier survival curves were generated to compare differences in overall survival between groups. In the present study, survival analyzes were restricted to overall survival (OS), whereas disease-free survival (DFS) and progression-free survival (PFS) were not evaluated.

Time-dependent receiver operating characteristic (ROC) curve analysis was used to compare the prognostic performance of AJCC-N, LNR and LODDs for predicting 1-, 3- and 5-year OS, and the corresponding time-dependent AUCs were calculated. Variables significant in univariable analyzes were entered into multivariable Cox proportional hazards models to identify independent prognostic factors. A LODDs-based nomogram was constructed based on the multivariable Cox model, and its performance was assessed using calibration curves, time-dependent C-index analysis and decision curve analysis. Time-dependent C-index values were calculated at 12, 24, 36, 48 and 60 months. A two-sided P value < 0.05 was considered statistically significant.

To address the potential influence of inadequate lymph node evaluation in the SEER cohort, sensitivity analyzes were performed among SEER patients with ≥16 examined lymph nodes. In this subgroup, Kaplan–Meier survival curves were generated for AJCC-N, LNR and LODDs categories, and time-dependent C-index values at 12, 24, 36, 48 and 60 months were calculated to compare the discriminatory performance of the three nodal staging systems.

### Ethics statement

The retrospective study involving human participants was reviewed and approved by the Ethics Committee of the First Affiliated Hospital of Hainan Medical University. In accordance with national regulations and institutional policies, informed consent was waived because anonymized archival data were used and no new interventions were introduced.

## Results

### Baseline characteristics

According to the inclusion and exclusion criteria, 4,054 patients in the SEER cohort were finally included. Among them, 2,838 were assigned to the training set and 1,216 to the internal validation set. In addition, 383 patients from the First Affiliated Hospital of Hainan Medical University were included in the external validation set.

Baseline demographic and clinicopathological characteristics of the three cohorts are summarized in **Table 1**. The median numbers of examined lymph nodes were 25 (IQR, 19–33), 25 (IQR, 19–34), and 21 (IQR, 17–28) in the training, internal validation, and external validation cohorts, respectively. The proportions of patients with ≥16 examined lymph nodes were 73.2%, 74.2%, and 68.4%, respectively. The median numbers of positive lymph nodes were 1 (IQR, 0–6), 1 (IQR, 0–6), and 1 (IQR, 0–5), and the median numbers of negative lymph nodes were 21 (IQR, 16–29), 22 (IQR, 16–30), and 18 (IQR, 13–25), respectively.

**Table 1:** Comparison of baseline demographic and clinical characteristics of gastric cancer patients across the three datasets.

Characteristics	Training set	INT validation set	EXT validation set
n	2838	1216	383
Age, median (IQR)	65 (57, 72)	65 (56, 72)	60 (52, 67)
Sex, n (%)			
Male	1768 (62.3%)	773 (63.6%)	257 (67.1%)
Female	1070 (37.7%)	443 (36.4%)	126 (32.9%)
Race, n (%)			
White	1852 (65.7%)	800 (66.1%)	0 (0%)
Black	357 (12.7%)	131 (10.8%)	0 (0%)
American Indian/Alaska Native	27 (1%)	8 (0.7%)	0 (0%)
Asian or Pacific Islander	582 (20.7%)	272 (22.5%)	383 (100%)
TNM stage (AJCC8), n (%)			
1	854 (30.1%)	397 (32.6%)	90 (23.5%)
2	914 (32.2%)	363 (29.9%)	93 (24.3%)
3	1070 (37.7%)	456 (37.5%)	200 (52.2%)
4	0 (0%)	0 (0%)	0 (0%)
T stage, n (%)			
T1	724 (25.5%)	324 (26.6%)	76 (19.8%)
T2	374 (13.2%)	157 (12.9%)	50 (13.1%)
T3	1091 (38.4%)	465 (38.2%)	51 (13.3%)
T4	649 (22.9%)	270 (22.2%)	206 (53.8%)
N stage, n (%)			
N0	1308 (46.2%)	572 (47.0%)	143 (37.3%)
N1	504 (17.8%)	210 (17.3%)	68 (17.8%)
N2	461 (16.1%)	178 (14.6%)	78 (20.4%)
N3a	369 (13.0%)	176 (14.5%)	60 (15.7%)
N3b	196 (6.9%)	80 (6.6%)	34 (8.8%)
Examined lymph nodes, median (IQR)	25 (19, 33)	25 (19, 34)	21 (17, 28)
Positive lymph nodes, median (IQR)	1 (0, 6)	1 (0, 6)	1 (0, 5)
Negative lymph nodes, median (IQR)	21 (16, 29)	22 (16, 30)	18 (13, 25)
ELNs ≥16, n (%)	2,077 (73.2%)	902 (74.2%)	262 (68.4%)
Tumor Size (mm), median (IQR)	37 (20, 60)	35 (20, 55)	35 (20, 55)
LNR, median (IQR)	0.041 (0, 0.234)	0.033 (0, 0.222)	0.093 (0, 0.440)
LODDs, median (IQR)	-1.19 (-1.58, -0.48)	-1.27 (-1.59, -0.52)	-0.85 (-1.46, -0.10)

### Correlation among AJCC-N, LNR, and LODDs

**Figure 1:**
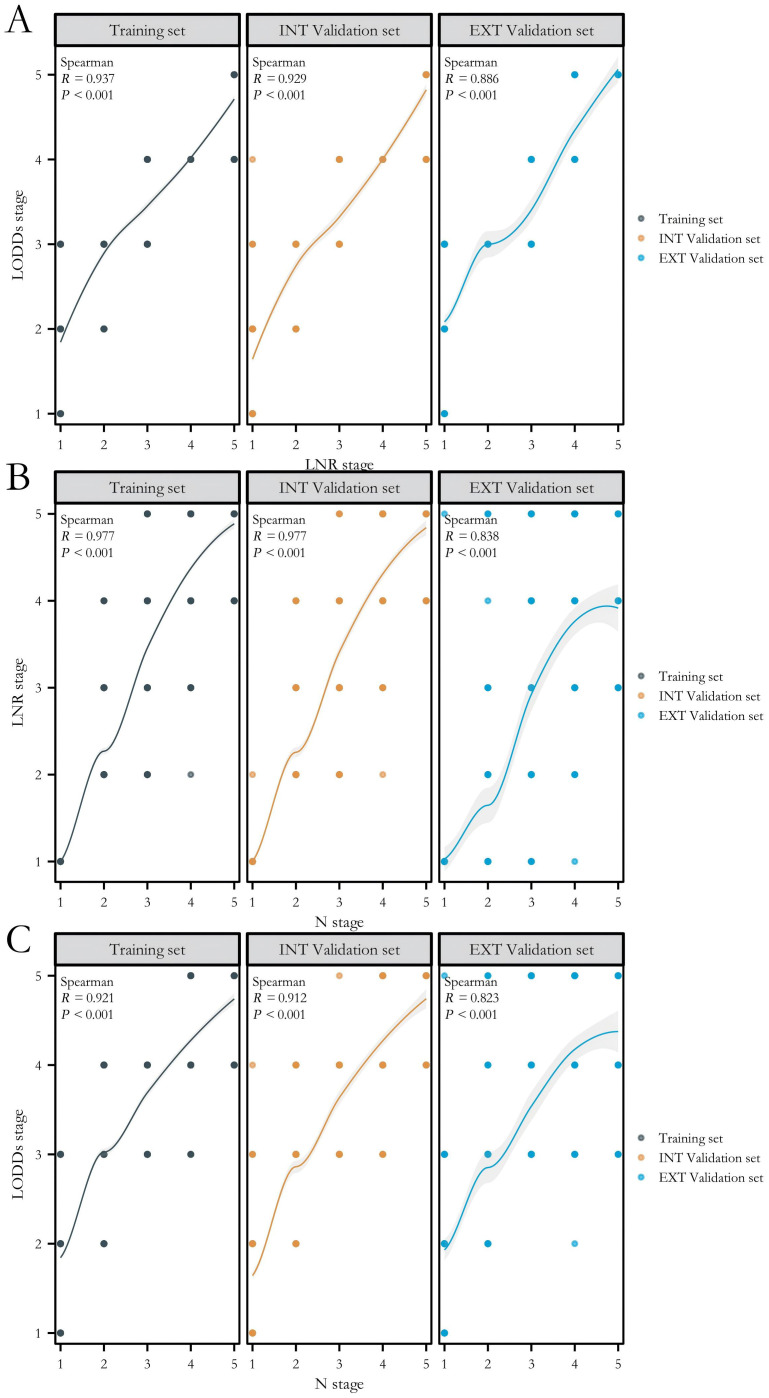
Correlation analysis among three lymph node staging methods in different datasets. **(A)** LODDs vs. LNR. **(B)** LNR vs. AJCC-N. **(C)** LODDs vs. AJCC-N.

Spearman correlation analysis was performed in the training, internal validation and external validation sets to assess associations among AJCC-N stage, LNR categories and LODDs categories using R software. In all three datasets, AJCC-N, LNR and LODDs were strongly and positively correlated with each other (P < 0.001; **Figure 1**).

### Survival according to the three nodal staging systems

**Figure 2:**
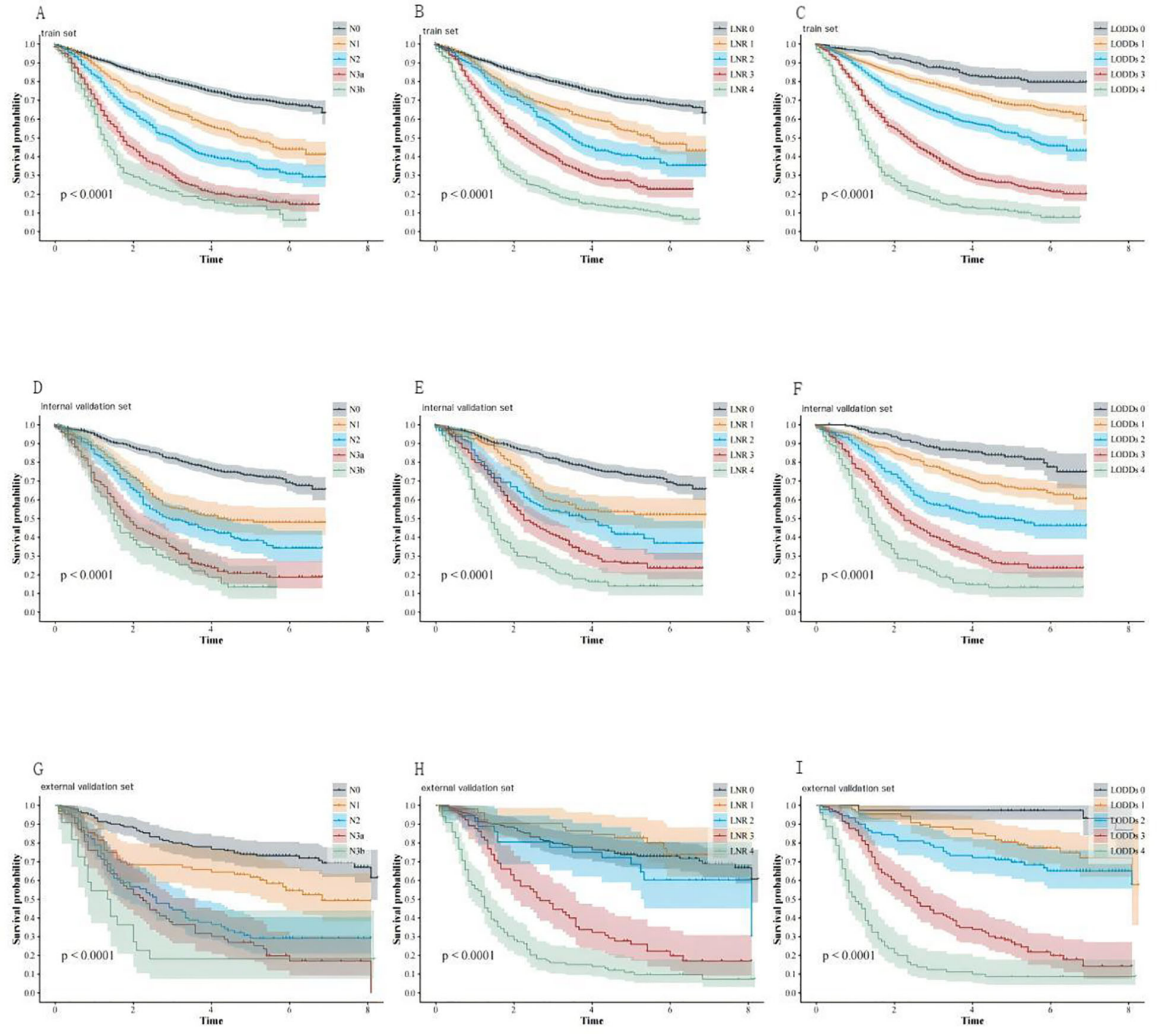
Survival curves for three lymph node staging methods in different cohorts. **(A–C)** Training set. **(D–F)** Internal validation set. **(G–I)** External validation set. Higher nodal stages were associated with progressively worse survival in all three systems.

X-tile software was used only in the training set to identify the optimal cut-off values for LNR and LODDs. The cut-off values for LNR were 0, 40% and 60%, and the cut-off values for LODDs were −1.5, −0.6 and 0.2. These cut-off values were fixed *a priori* after derivation in the training set and were applied without re-estimation to the internal validation cohort, external validation cohort and ELNs ≥16 sensitivity subgroup. Based on these values, all patients were categorized accordingly. LNR was divided into five groups (LNR0, LNR1, LNR2, LNR3, LNR4) from lower to higher values. LODDs was similarly divided into five groups (LODDs0, LODDs1, LODDs2, LODDs3, LODDs4). Kaplan–Meier analysis showed significant survival differences across AJCC-N, LNR, and LODDs categories in the training, internal validation, and external validation cohorts (all log-rank P < 0.0001; **Figure 2**). In all three systems, survival worsened progressively with increasing nodal stage. Notably, the separation of survival curves appeared clearer for LODDs, suggesting better discriminatory ability for prognostic stratification.

### Time-dependent ROC analysis

**Figure 3:**
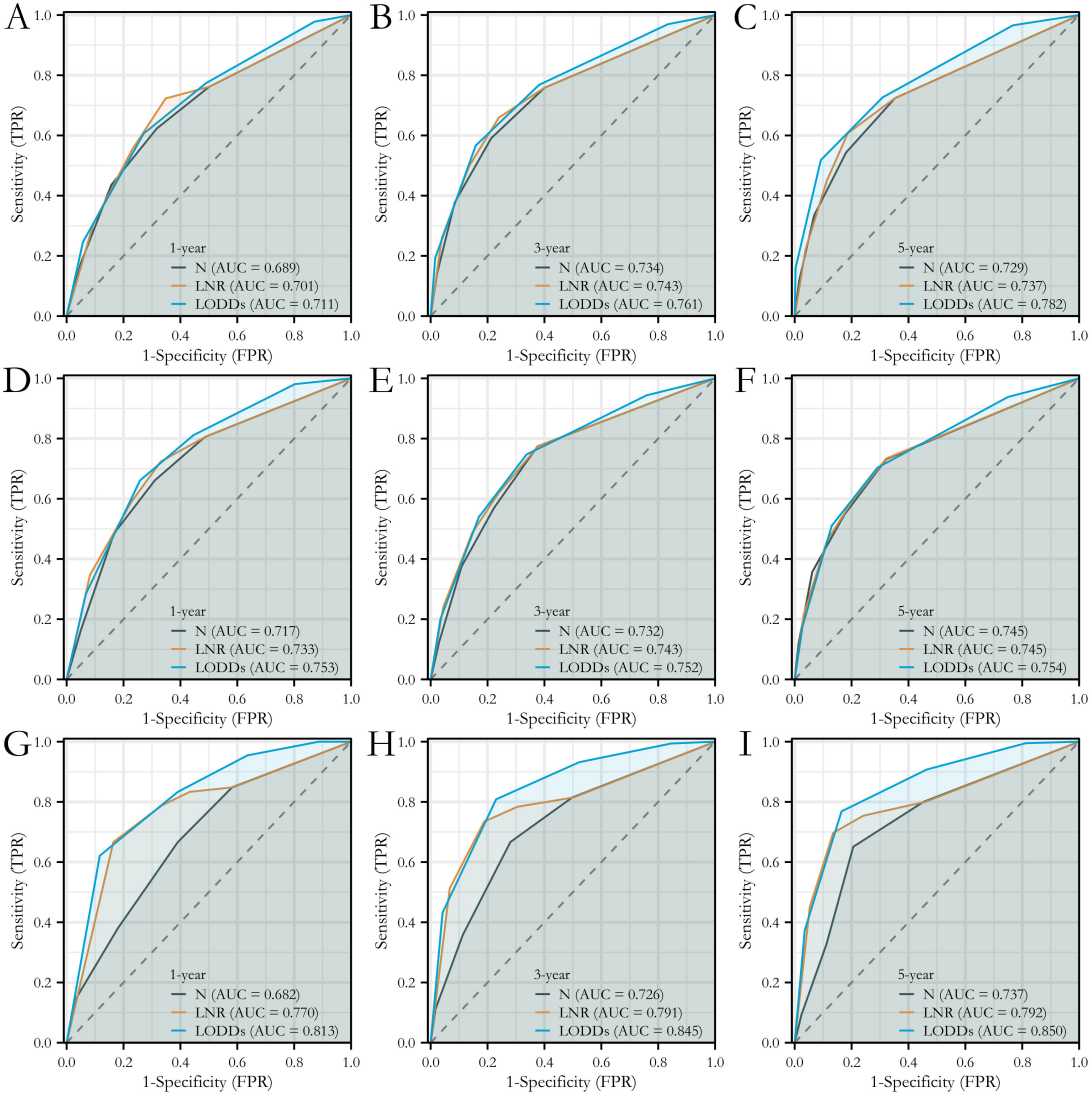
Time-dependent ROC curves for three lymph node staging methods across different cohorts. **(A–C)** Time-dependent ROC curves for 1-, 3-, and 5-year survival in the training set. **(D–F)** Time-dependent ROC curves for 1-, 3-, and 5-year survival in the internal validation set. **(G–I)** Time-dependent ROC curves for 1-, 3-, and 5-year survival in the external validation set. LODDs generally showed the highest AUC values.

Time-dependent ROC curves were plotted to compare the predictive performance of AJCC-N, LNR, and LODDs for 1-, 3-, and 5-year overall survival. In the training and internal validation cohorts, both LNR and LODDs showed higher time-dependent AUC values than AJCC-N, with LODDs performing slightly better overall. In the external validation cohort, LODDs also showed favorable time-dependent AUC values for 1-, 3- and 5-year survival prediction (**Figure 3**). These findings suggest that LODDs provided the best overall prognostic discrimination among the three nodal staging systems.

### Sensitivity analysis in SEER patients with ≥16 examined lymph nodes

Because anatomical D2 lymphadenectomy could not be directly identified in the SEER database, we performed sensitivity analyzes restricted to SEER patients with ≥16 examined lymph nodes. This subgroup included 2,979 patients, comprising 2,077 patients from the training set and 902 patients from the internal validation set.

Kaplan–Meier analysis showed significant survival differences across AJCC-N, LNR and LODDs categories in this subgroup, with all log-rank P values < 0.0001 (**Supplementary Figure 1**). Time-dependent C-index analysis further showed that LODDs consistently achieved higher C-index values than AJCC-N and LNR at 12, 24, 36, 48 and 60 months (**Supplementary Figure 2**). The C-index values for AJCC-N were 0.698, 0.698, 0.688, 0.683 and 0.678 at 12, 24, 36, 48 and 60 months, respectively. The corresponding values were 0.700, 0.694, 0.700, 0.694 and 0.691 for LNR, and 0.730, 0.731, 0.722, 0.717 and 0.714 for LODDs. These findings indicate that the prognostic performance of LODDs remained stable among SEER patients with adequate lymph node evaluation.

### Univariable and multivariable analyzes of overall survival

To identify independent predictors of overall survival (OS) in patients with gastric adenocarcinoma without distant metastasis, we first performed univariable analyzes and then included variables with P < 0.05 in multivariable Cox regression models. Because the present study focused on OS as the primary endpoint, DFS and PFS were not analyzed.

**Figure 4:**
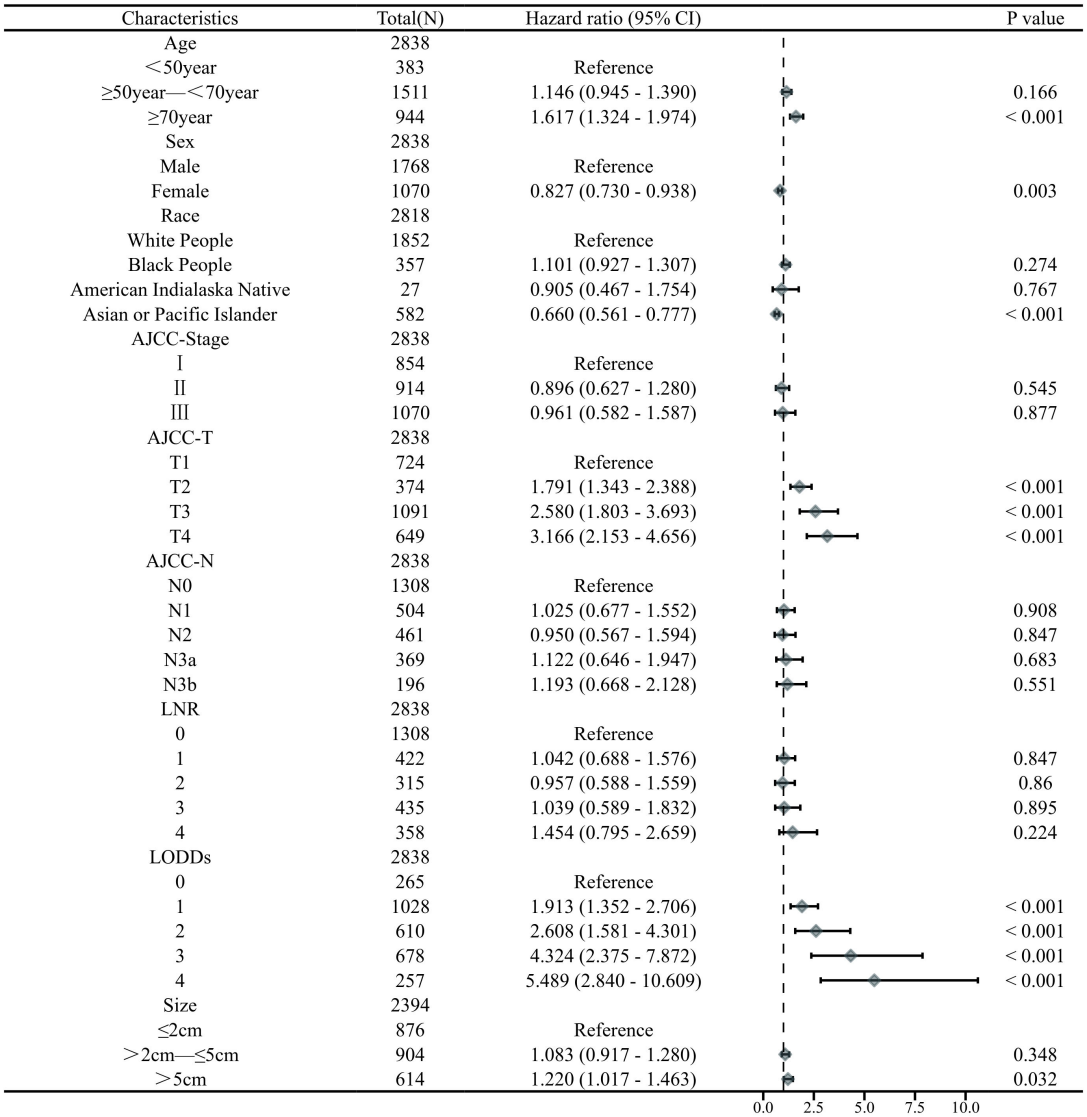
Univariable and multivariable analyzes of factors associated with overall survival in the training set.

After adjustment for potential confounders, sex, age, race, T stage, LODDs and tumor size remained significantly associated with OS (P < 0.05 for all). These variables were therefore considered independent prognostic factors (**Figure 4**).

### Nomogram construction and calibration

**Figure 5:**
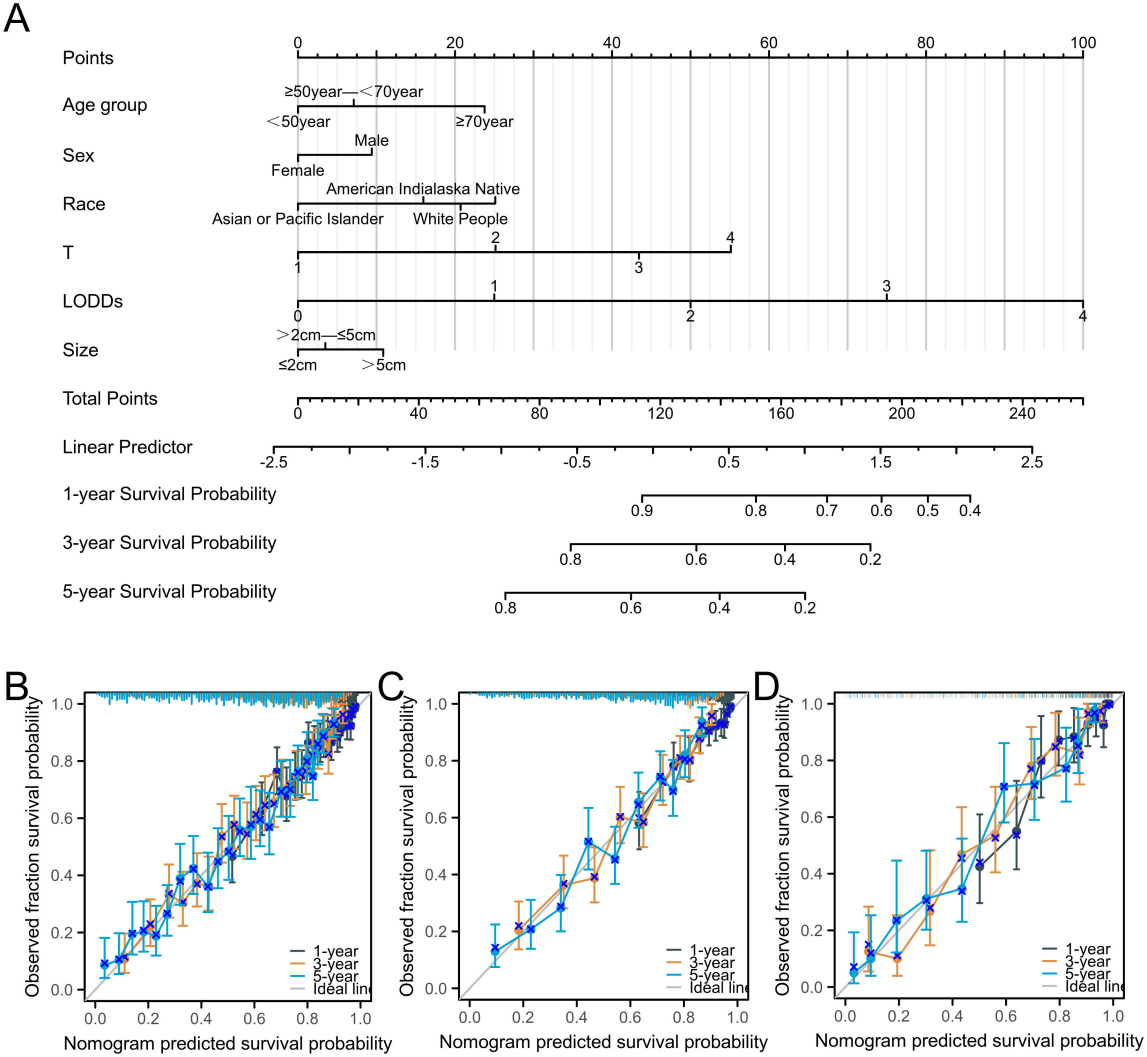
Nomogram and calibration curves based on multivariable analysis. **(A)** Nomogram constructed from independent prognostic factors. **(B)** Calibration curves for the training set. **(C)** Calibration curves for the internal validation set. **(D)** Calibration curves for the external validation set. The calibration plots show good agreement between predicted and observed survival probabilities.

Based on the results of multivariable Cox regression, we used the rms package in R to derive regression coefficients and corresponding point allocations for age, sex, race, AJCC-T stage, LODDs category and tumor size. These factors were combined to construct a nomogram that visually displays the contribution of each predictor to the estimated outcome (**Figure 5A**).

To evaluate the calibration of the nomogram in predicting prognosis in gastric adenocarcinoma, we generated calibration curves and used bootstrap resampling (1,000 repetitions) for internal validation to correct potential overfitting (**Figure 5**). The calibration plots in the training, internal validation, and external validation cohorts showed generally good agreement between predicted and observed 1-, 3-, and 5-year survival probabilities, with most curves lying close to the ideal reference line. Bootstrap validation further supported the robustness of the model.

**Figure 6:**
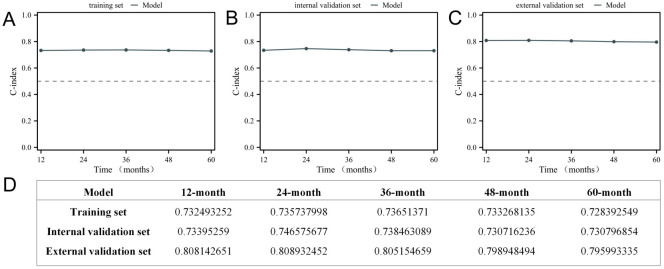
Time-dependent C-index of the LODDs-based nomogram across the training, internal validation and external validation cohorts. **(A–C)** Time-dependent C-index curves of the LODDs-based nomogram in the training set, internal validation set and external validation set at 12, 24, 36, 48 and 60 months. **(D)** Corresponding C-index values at each time point. The dashed horizontal line indicates a C-index of 0.5.

The discriminative ability of the LODDs-based nomogram was further assessed using time-dependent C-index analysis. Across 12 to 60 months, the C-index values ranged from 0.728 to 0.737 in the training set, from 0.731 to 0.747 in the internal validation set, and from 0.796 to 0.809 in the external validation set (**Figure 6**). These findings indicate that the nomogram had stable discriminatory performance across the development and validation cohorts.

### Decision curve analysis

**Figure 7:**
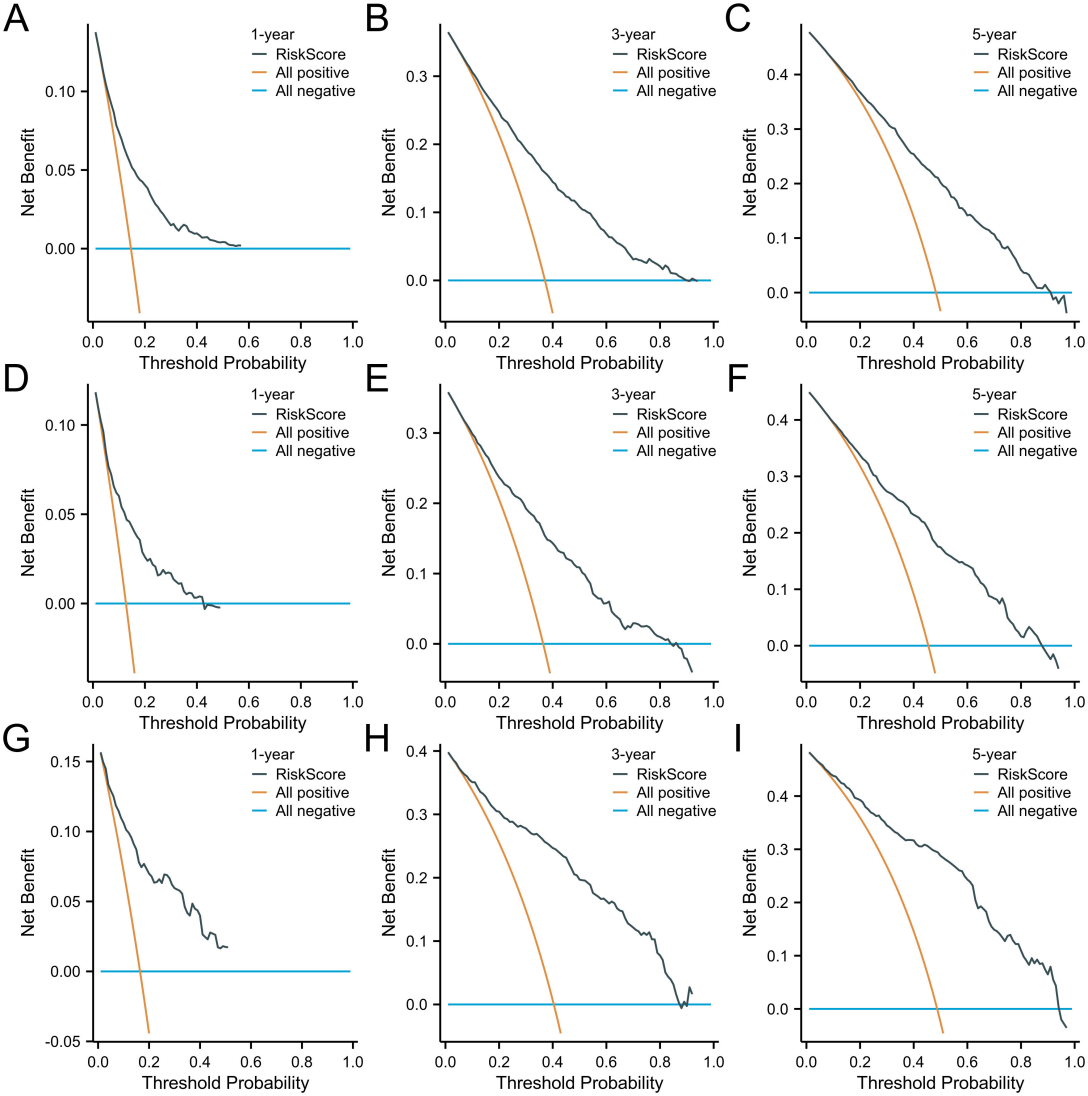
Decision curve analysis in different cohorts. **(A–C)** Training set for 1-, 3-, and 5-year survival prediction. **(D–F)** Internal validation set for 1-, 3-, and 5-year survival prediction. **(G–I)** External validation set for 1-, 3-, and 5-year survival prediction. The nomogram provided favorable net benefit across a broad range of threshold probabilities.

To further examine the clinical utility of the nomogram, we performed decision curve analysis. DCA evaluated the net benefit associated with decisions based on the nomogram across a range of threshold probabilities (**Figure 7**).

The analyzes showed that the nomogram provided a higher net benefit than the treat-all and treat-none strategies across a broad range of threshold probabilities in all three cohorts, indicating its potential clinical usefulness for individualized prognostic assessment and decision-making.

## Discussion

Gastric cancer remains one of the main causes of cancer-related death worldwide, and lymph node status is a key determinant of prognosis. Nodal metastasis is common in patients with gastric cancer and is closely associated with poorer outcomes (18). Studies have shown that both the depth of tumor invasion and the extent of nodal involvement are important predictors of prognosis (18). Although the AJCC TNM system, particularly the N category, is widely used for prognostic evaluation, its accuracy and discriminatory ability have been questioned (19, 20). To improve nodal staging, several alternative or complementary methods have been proposed. Among these, the metastatic lymph node ratio (LNR) has been recognized as an important prognostic factor, and several studies have reported that it provides better prognostic discrimination than the conventional pN classification (21, 22).

LODDs has emerged as a promising nodal staging index and has demonstrated favorable performance in several malignancies. In gallbladder cancer, for example, LODDs-based nomograms have shown good predictive ability for 1-, 3- and 5-year survival (23). In esophageal cancer, LODDs also demonstrated strong predictive performance (24). Similarly, in colorectal cancer, LODDs has been validated as an independent prognostic factor (25).

Recent evidence has further supported the prognostic value of LODDs in gastric cancer, including a 2023 systematic review and meta-analysis (26) and several LODDs-based nomogram studies in selected populations such as T1 gastric cancer (27), elderly gastric adenocarcinoma (28), late-onset gastric adenocarcinoma (29) and gastric signet ring cell carcinoma (30). Compared with these studies, our work focused on surgically treated gastric adenocarcinoma with available lymph node evaluation, directly compared AJCC-N, LNR and LODDs, included external institutional validation and added ELNs ≥16 sensitivity analyzes. Therefore, this study provides additional comparative, externally validated and ELN-restricted evidence for the prognostic utility of LODDs.

In our study, we compared three approaches reflecting different perspectives: a conventional, clinically familiar system (AJCC-N), a ratio-based measure (LNR) and a mathematically transformed index (LODDs). We then integrated multiple independent predictors into a visual nomogram to provide a practical, individualized prognostic tool for clinicians. Our results suggest that LODDs offers the best discrimination among the three nodal staging systems. The nomogram that includes LODDs along with age, sex, race, T stage and tumor size demonstrated good discriminative ability and calibration in both internal and external validation cohorts. Decision curve analysis further indicated that this model may provide net clinical benefit within a wide range of reasonable threshold probabilities. The superior performance of LODDs observed in this study is consistent with previous reports. By combining the numbers of positive and negative lymph nodes using a logarithmic transformation, LODDs reduces the dependence of nodal assessment on the total number of examined nodes and may better capture subtle differences in nodal burden. Importantly, when the number of positive nodes is zero, LODDs can still differentiate patients according to the number of examined lymph nodes, whereas traditional N categories and LNR cannot distinguish among node-negative patients in this way. As a continuous variable, LODDs may therefore better reflect small changes in metastatic burden, which could underlie its improved prognostic performance.

An important methodological consideration of the present study is the heterogeneity of lymphadenectomy in population-based registry data. D2 lymphadenectomy is an anatomically defined surgical procedure based on the extent of nodal station dissection, whereas SEER does not provide sufficiently detailed station-level operative information to reliably distinguish D1, D1+ and D2 lymphadenectomy. Therefore, in the revised manuscript, D2 lymphadenectomy was confirmed only in the institutional validation cohort through operative records and pathological reports. For the SEER cohort, examined lymph node count was used to evaluate the adequacy of lymph node assessment rather than to define D2 lymphadenectomy. In sensitivity analyzes restricted to SEER patients with ≥16 examined lymph nodes, AJCC-N, LNR and LODDs all showed significant survival stratification, and LODDs maintained the highest time-dependent C-index values across 12 to 60 months. These findings suggest that the prognostic value of LODDs was not solely driven by patients with insufficient lymph node evaluation.

The nomogram constructed in this study incorporates six independent predictors—age, sex, race, T stage, LODDs and tumor size—and showed better predictive accuracy than any single staging system alone. DCA suggested that the nomogram may support individualized clinical decision-making for patients with gastric adenocarcinoma without distant metastasis, help optimize allocation of medical resources and improve the cost-effectiveness of treatment strategies.

Overall, current evidence suggests that lymph node staging in gastric cancer has gradually evolved from simple counting-based systems toward more refined quantitative models. Although AJCC-N remains the most widely used and clinically familiar system, its performance may be influenced by the number of examined lymph nodes. LNR partly addresses this issue by incorporating the proportion of metastatic nodes, whereas LODDs further integrates information from both positive and negative lymph nodes and may provide additional discriminatory value, particularly in patients with limited nodal yield or node-negative disease. Our findings add to this growing body of evidence by showing that LODDs achieved the best overall prognostic performance among the three evaluated systems. Future studies should focus on multicenter prospective validation, broader external validation, and integration of additional clinicopathological or molecular variables to further improve individualized prognostic assessment.

This study has several limitations. First, although D2 lymphadenectomy was confirmed in the institutional validation cohort through operative records and pathological reports, the anatomical extent of lymphadenectomy could not be directly determined in the SEER cohort. Therefore, the SEER cohort should not be interpreted as a uniformly D2-dissected population. We used examined lymph node count as an indicator of lymph node evaluation adequacy and performed sensitivity analyzes in patients with ≥16 examined lymph nodes. However, examined lymph node count is not equivalent to D2 lymphadenectomy and may still introduce selection bias or residual confounding. Second, the external validation cohort was derived from a single center, which may limit the generalizability of the findings. Third, only overall survival was analyzed because DFS and PFS were not available. Further multicenter prospective studies with clearly defined lymphadenectomy extent are needed to validate our findings.

In addition, although X-tile-derived cut-offs were determined only in the training cohort and applied unchanged to the validation cohorts, categorizing continuous variables such as LNR and LODDs may still cause information loss and potential residual overfitting.

## Conclusion

Among surgically treated patients with gastric adenocarcinoma and available lymph node evaluation, LODDs provided a sensitive reflection of metastatic tumor burden and showed favorable prognostic performance compared with AJCC-N and LNR. In SEER sensitivity analyzes restricted to patients with ≥16 examined lymph nodes and in the institutional cohort with confirmed D2 lymphadenectomy, the prognostic value of LODDs remained generally consistent. These findings provide additional evidence supporting LODDs as a complementary nodal staging indicator rather than a replacement for established staging systems. The LODDs-based nomogram may serve as a useful tool for individualized prognostic assessment, although further prospective validation in cohorts with well-defined lymphadenectomy extent is warranted.

## Data Availability

The original contributions presented in the study are included in the article/**Supplementary Material**. Further inquiries can be directed to the corresponding author.

## Supplementary material

Supplementary Figure 1 Kaplan–Meier survival curves according to **(A)** AJCC-N; **(B)** LNR; and **(C)** LODDs categories in SEER patients with ≥16 examined lymph nodes. Log-rank P values are shown.

Supplementary Figure 2 Time-dependent C-index curves of **(A)** AJCC-N; **(B)** LNR; and **(C)** LODDs in SEER patients with ≥16 examined lymph nodes. D, C-index values at 12, 24, 36, 48 and 60 months.
