# Supplementary figures and images for: Optimizing gastric cancer treatment: the role of LODDs in lymph node staging
Source: Front Oncol. 2026 Jun 9;16:1828429. doi: 10.3389/fonc.2026.1828429 (PMC13286846; doi:10.3389/fonc.2026.1828429)

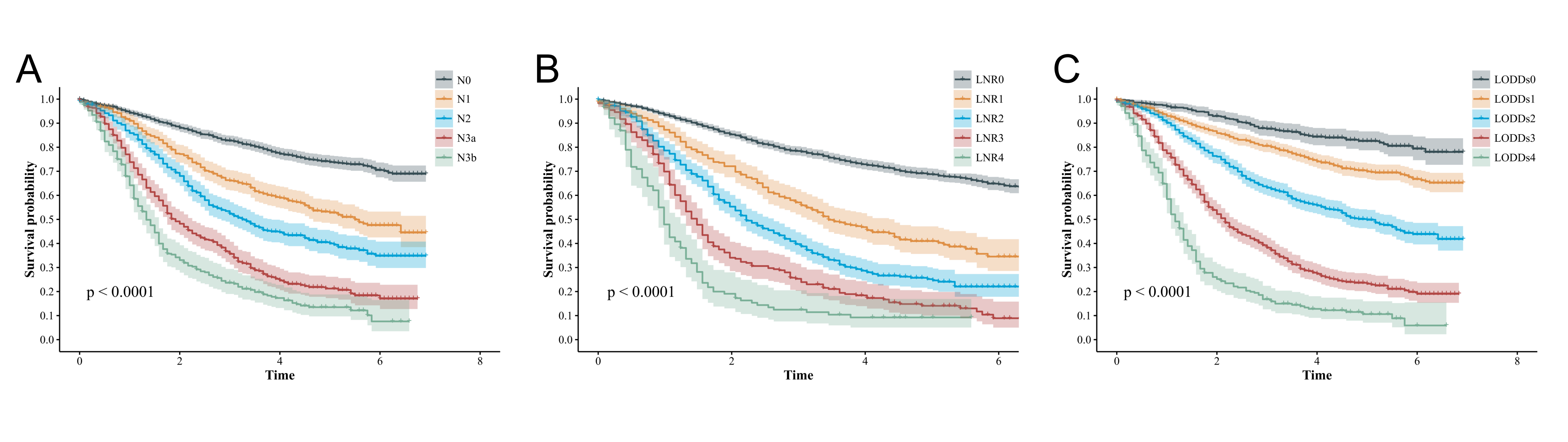

Supplement: Supplementary Figure 1 — Kaplan–Meier survival curves according to (A) AJCC-N; (B) LNR; and (C) LODDs categories in SEER patients with ≥16 examined lymph nodes. Log-rank P values are shown. [file Image1.png]

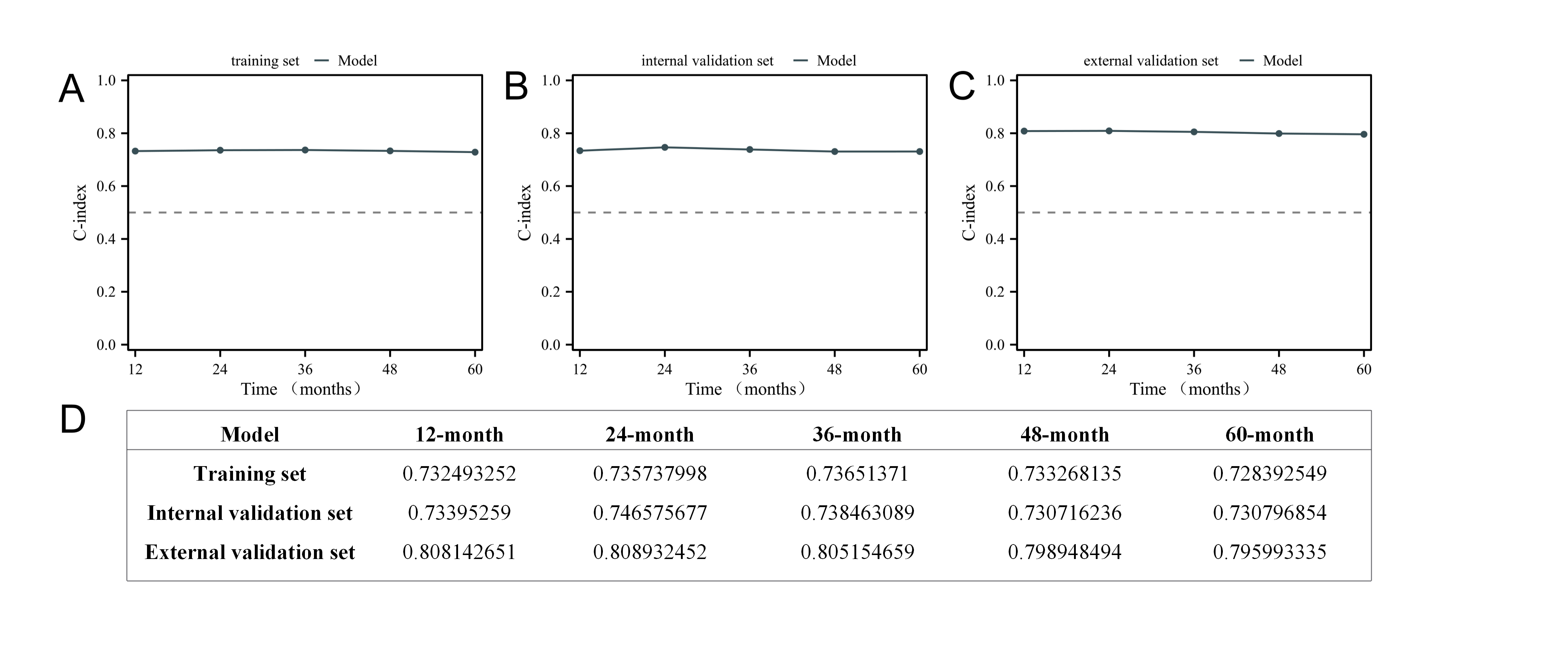

Supplement: Supplementary Figure 2 — Time-dependent C-index curves of (A) AJCC-N; (B) LNR; and (C) LODDs in SEER patients with ≥16 examined lymph nodes. D, C-index values at 12, 24, 36, 48 and 60 months. [file Image2.png]
